# Management of awake bruxism: a systematic review

**DOI:** 10.1186/s12903-026-07856-z

**Published:** 2026-02-24

**Authors:** David A. Graham, Anna Lövgren, Birgitta Häggman-Henrikson, Christopher C. Peck, Daniele Manfredini, Ovidiu Ionut Saracutu

**Affiliations:** 1 Faculty of Medicine and Health, School of Dentistry, University of Sydney, Sydney, NSW Australia; 2 Department of Odontology, Orofacial Pain and Jaw Function, Faculty of Medicine, Umeå University, Umeå, 901 87 Sweden; 3 Department of Orofacial Pain and Jaw Function, Faculty of Odontology, Malmö University, Malmö, Sweden; 4 Faculty of Dentistry, National University of Singapore, Singapore, Singapore; 5 Department of Medical Biotechnologies, University of Siena, Siena, Italy

**Keywords:** Awake bruxism, Behavior therapy, Biofeedback, Cognitive-behavioural therapy, Ecological momentary assessment

## Abstract

**Background:**

Awake bruxism is a masticatory muscle activity reported by approximately one in four individuals in the general population. Bruxism can have various manifestations such as tooth clenching, grinding or bracing, with different possible clinical consequences, ranging from tooth wear and fractures of dental restorations to orofacial pain related to the musculoskeletal overload. However, no official guidelines exist on the management of awake bruxism. The aim of this systematic review was to review published literature on management of awake bruxism.

**Methods:**

The review followed the PRISMA guidelines. After registration in Prospero (CRD42022351309), an electronic literature search was conducted in Medline, Embase, Scopus, LILACS, CINAHL, OpenGrey, Trip Medical, and Google on 30 August 2023 to identify articles on management of awake bruxism in participants aged 13 years and above. The electronic search was complemented with a hand search of reference lists. The review process was managed in Covidence. Two researchers independently screened titles, abstracts, and full-text articles according to the inclusion criteria which required the studies to be clinical studies involving teenagers or adults, written in English, and presented as randomized controlled trials, non-randomized controlled trials, reports or trials, or case series reports. Risk of bias was assessed using the Newcastle-Ottawa Scale, the Joanna Briggs Institute Critical Appraisal Checklist for case-series, or the RoB 2 scale, depending on study design.

**Results:**

After screening of 4358 abstracts, 210 articles were assessed in full text and nine studies, with 165 participants, were included and summarized in a qualitative synthesis. The most common reason for exclusion was having the wrong study design. In addition, 30 articles were excluded because of not evaluating an awake bruxism population. In the included studies, most evaluated strategies fall within the broad category of cognitive behavioural therapy. The included studies, despite varying risks of bias, generally suggested positive effects of biofeedback, guided music listening, habit reversal, medication, reminder prompts, and counselling with self-management. Given the heterogeneity of included studies, meta-analysis was deemed not appropriate.

**Conclusion:**

The available evidence is limited but still suggests benefits of a behavioural approach to managing the frequency and consequences of awake bruxism.

**Supplementary Information:**

The online version contains supplementary material available at 10.1186/s12903-026-07856-z.

## Background

In 2025, an international panel of experts formulated a general definition of bruxism: a repetitive jaw-muscle activity, characterized by clenching or grinding the teeth and/or bracing or thrusting the mandible, that can have two distinct circadian manifestations, one during sleep, i.e., sleep bruxism, and one during wakefulness, i.e., awake bruxism (AB), with differences in aetiology as well as clinical consequences. One of the main points of the consensus statement was the proposal that bruxism must be viewed as a masticatory muscle activity rather than a disorder, with its clinical relevance depending on the presence and severity of the clinical consequences. Additionally, the updated 2025 consensus paper on bruxism redefined AB as a masticatory muscle activity during wakefulness that is characterized by repetitive or sustained tooth contact and/or by bracing or thrusting the mandible [1]. This implies that AB can have various manifestations with possible clinical consequences, ranging from tooth wear [2] to musculoskeletal orofacial pain related to the mechanical overload [3, 4].

Recent evidence suggests that approximately one in four individuals in the general population reports AB[5]. However, the prevalence varies considerably depending on the type of population studied, geographical areas as well as clinical settings [6], and may reach 40–50% of patients with systemic conditions and/or temporomandibular disorders (TMDs) [7–9]. Indeed, most studies are based on the patient’s self-report of bruxism, the validity of which has been questioned [10, 11] due to the potentially arbitrary dichotomization of patients into two broad categories of bruxers and non-bruxers. AB prevalence data should therefore be interpreted with caution, given that the prevalence of AB strictly depends on the assessment methods used [12, 13].

In this context, the recent reconceptualization of bruxism has underlined the importance of assessing bruxism not only in terms of its prevalence or incidence, but also in terms of the frequency of the specific masticatory muscle activities [14]. Instrumentally driven assessment of bruxism, based on the principles of the Ecological Momentary Assessment (EMA) [14], has shown that a certain amount of bruxism-related masticatory muscle activity can be expected in all individuals, and that the clinical relevance is related to the frequency and intensity of the events [15, 16]. Furthermore, the advent of portable surface electromyographic (EMG) devices that can record 24-hour jaw muscle activity has provided new possibilities to monitor both AB and sleep bruxism in the natural environment [17–19].

Currently available knowledge suggests that the frequency of AB is largely associated, even in healthy pain-free individuals, with the degree of psychological distress, especially anxiety and depression [20–24]. Other factors have also been associated with AB, such as genetics [25] and the use of certain substances, such as tobacco [26], drugs [27], and alcohol [28]. However, the effects of these substances are likely attributable to their psychotropic effects on the dopaminergic modulation, arousal regulation, and stress reactivity at the level of the central nervous system (CNS) [29], confirming the role of the brain in determining AB frequency.

From a clinical perspective, patients often seek dental consultation after the appearance of AB’s consequences rather than for its causes, often without even being aware of their own habit of clenching their teeth or bracing their mandible during wakefulness [30]. In this regard, the main reason why patients with a high frequency of bruxism come to the attention of dentists can be due to aesthetic needs to restore worn dentition, but also due to different types of TMDs and other pain conditions such as headache, migraine, neck pain, dentine hypersensitivity, or temporomandibular joint dysfunction [31–35]. In such clinical situations, dentists should know the above-mentioned list of potential aetiological factors because they ideally call for specific strategies for reducing the patient’s AB frequency, mitigating and reducing the effect of AB on the stomatognathic system, and preventing the relapse of pain or the failure of restorative treatment [36, 37].

In this scenario, it is not easy for clinicians to find official guidelines and approaches for the management of AB. Practitioners face important challenges when deciding what treatment to implement for AB management, or even when confirming that the patient has been reducing his/her frequency of AB. Such confusion is also a factor contributing to the often-described mis- or over-treatment of patients with bruxism and/or TMDs [38, 39]. In this regard, a recent study has revealed that up to 50% of dental practitioners propose occlusal adjustments as a treatment option to patients who report signs of bruxism [40]. Such an approach contradicts evidence-based recommendations because of the lack of role of occlusal factors in the aetiology of bruxism [41–43]. On the contrary, AB treatment approaches should target the aetiological and risk factors, as in the case of behavioural therapy [44] or electromyographic devices that provide a real-time biofeedback to the patient [45]. Over the years, numerous therapeutic approaches have been proposed for the management of AB[46]. These can be categorized into behavioural strategies (e.g. cognitive-behavioural therapy (CBT), biofeedback, mindfulness, EMA-assisted self-monitoring), pharmacological, instrumental and multidisciplinary strategies (including for example dentists, physiotherapists, and psychologists). Although there has been a number of published reviews on the topic, they have mostly focussed only on biofeedback treatment [47] and not systematically summarised all the existing strategies for managing AB.

The aim of this systematic review was to evaluate the evidence on management strategies for AB in both experimental and observational settings, among adults, with frequency of AB as the primary outcome, to provide practitioners with evidence-based recommendations for good clinical practice.

## Methods

A systematic review was undertaken following the PRISMA Statement guidelines [48] to answer the question “For teenage and adult humans (aged 13 and above), what are the reported strategies for the management of awake bruxism?” using the PICO framework: Population = “teenage and adult humans”, Intervention = “evaluated forms of management”, Comparator = “other interventions or no treatment”, and Outcome = “awake bruxism behaviour”. The study was registered with PROSPERO (registration number: CRD42022351309).

### Search strategies

The electronic search was performed in Medline, Embase, Scopus, LILACS, CINAHL, Trip Medical, OpenGrey, and Google from the inception of each database until 30 August 2022 and updated on 30 August 2023. In addition, the reference lists of the included articles were manually searched.

The search strategy and search string were generated around three search blocks, i.e., “awake”, “bruxism”, and “management”, along with the most frequently used synonyms for each of these words sought from the dental literature included in the string. See the Supplementary File for full details of all search strategies.

### Inclusion criteria

The included studies were clinical studies involving teenagers or adults, written in English, and in the form of randomized controlled trials, non-randomized controlled trials, reports or trials, and case series reports. The inclusion criteria, summarised as Population, Intervention, Comparison, Outcomes and study design (PICOs), are provided in Table 1.

**Table 1:** Criteria for inclusion (PICOs): population (P), intervention (I), comparison (C), outcomes (O) and study design (s)

Population	Teenagers (age ≥ 13 years) and adults
Intervention	Any intervention aimed at managing awake bruxism behaviour
Comparison	No treatment, placebo treatment, other interventions
Outcome	Awake bruxism behaviours
Study design	Randomized controlled trials, non-randomized controlled trials, case-control studies, case series

### Exclusion criteria

Studies limited to sleep bruxism, or children, or written in languages other than English were excluded.

### Study selection

All references from the search were collected in EndNote20 reference management software (Thompson Reuters, Philadelphia, PA., USA). Once duplicates had been removed, all references were imported into Covidence systematic review software (Veritas Health Innovation, Melbourne, Australia).

Two researchers (AL and DG) independently screened the references by title and abstract according to the criteria described above. Full-text versions were obtained for all studies deemed to possibly meet the inclusion criteria by at least one reviewer, or for which there were insufficient data in the title and abstract to make a clear decision.

### Full-text review and data extraction

Full-text assessment was conducted independently by the same two reviewers (AL and DG) with moderate inter-rater reliability (Cohen’s kappa 0.45) and any disagreements were resolved by discussion or by a third reviewer (BHH) if needed. Any excluded study was assigned to one of a predetermined set of reasons (see Supplementary File).

The following data were extracted from all included studies in Covidence by two independent reviewers (AL and DG) and reviewed by a third reviewer (BHH): first author, year, study setting, country, study design, aim, study sample with mean age range, interventions, outcomes, authors’ conclusion, comments.

### Quality/risk-of-bias assessment

The quality/risk of bias of the included studies was assessed independently by two reviewers (AL and DG) and reviewed by a third reviewer (BHH) using the Newcastle-Ottawa scale (NOS) [49] for case-series, case-control, and cohort studies, the Joanna Briggs Institute Critical Appraisal Checklist for case-series [50], and the RoB 2 scale for randomized controlled trials [51]. Using the RoB 2 scale, each study was assessed for bias in five domains, i.e., selection, performance, attrition, detection, and reporting, with three response options, i.e., low, unsure, or high. The overall risk of bias for a study usually corresponds to the worst risk in any of the domains; however, a study eliciting multiple “unsure” responses might be judged as having a high risk of overall bias.

Human Ethics and Consent to Participate declarations: not applicable.

## Results

### Literature searches

**Fig. 1:**
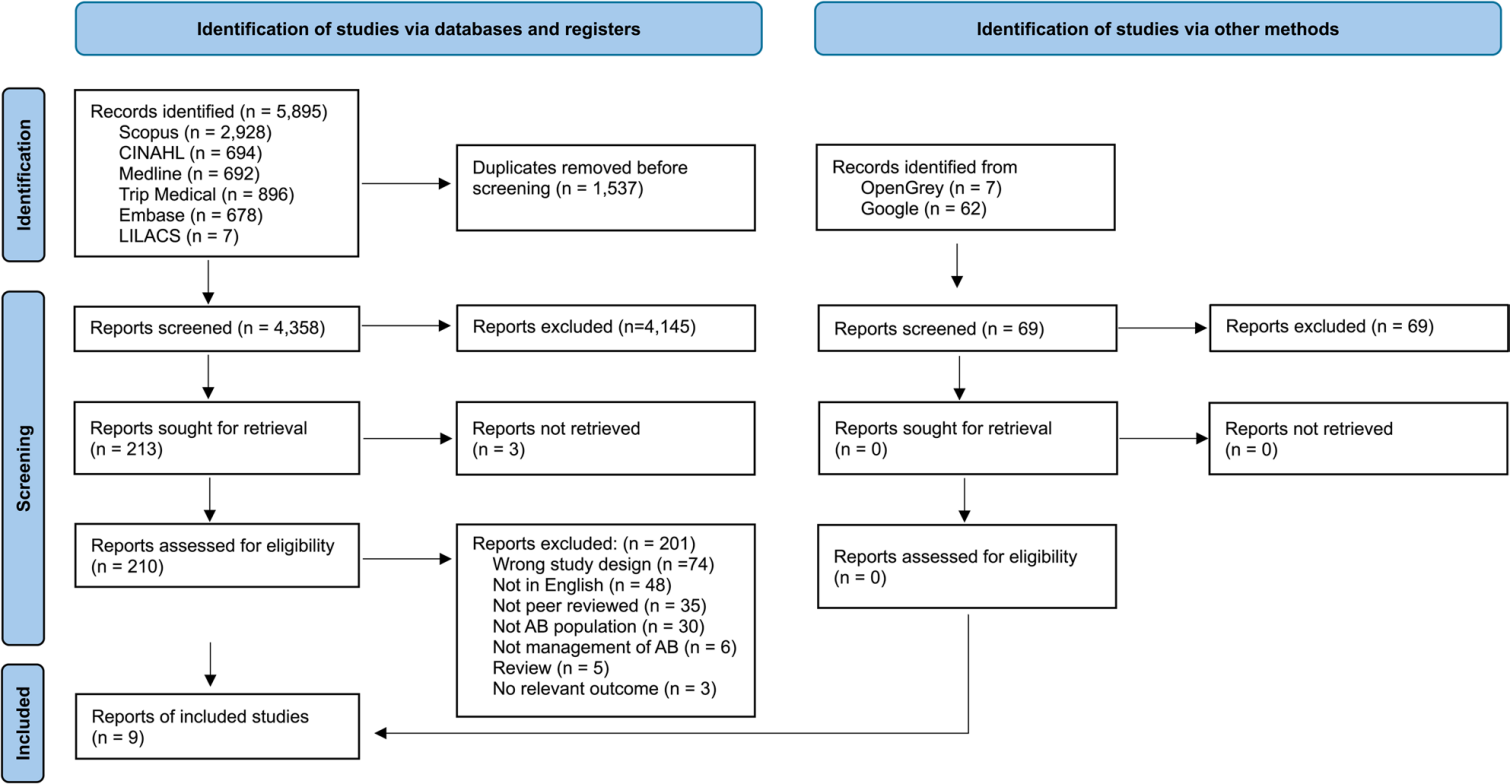
PRISMA flowchart of the included and excluded studies

In total, 5895 records were identified in databases and an additional 69 records by other sources (Fig. 1). After removal of 1537 duplicates, the reviewers independently screened 4358 abstracts from databases and 69 abstracts from other sources and 4214 of these were excluded. The full-text assessment of the remaining 210 articles led to the exclusion of 201 of them because they did not meet the inclusion criteria (Table 2 and Supplementary File). The most common reason for exclusion was having the wrong study design. Based on the inclusion/exclusion criteria, nine studies with 165 participants were included in the review.

**Table 2:** Main reason for exclusion at full-text level (*n* = 204)

**First author, year**	**Main reason for exclusion**
Ackerman 1966, Attanasio 1997, Attanasio 2000, Ayer 1975, Benk 2004, Bidaki 2022, Blanchet 2010, Blount 1982, Boxley 2017, Bracci 2022, Brown 1999, Brown 2022, Cannistraci 1976, Cannistraci 1987, Cherasia 1986, Dahl 1987, DuPontJr 2006, Ellement 2021, Evans 2011, Gittelson 2005, Goldstein 2017a, Goldstein 2017b, Gouw 2017, Graf 1969, Greenwald 1968, Guaita 2016, Guevara 1998, Gurusidheshwar 2004, Gutman 1975, Heller 1973, Hennessy 2022, Ilovar 2014, Ingersoll 1952, Kaner 1952, Keith 1978, Kowacs 2021, Krattenmaker 2017, Kumar 2018, Kwon 2019, Lang 2013, Leib 1996, Leung 1991, Lobbezoo 2008, Malcmacher 2013, Mathew 2020, Meklas 1971, MuñozLora 2019, Myers 1990, Nash 2004, Nissani 2000, Okeson 1983, Ondo 2011, Palumbo 2007, Pina-Escudero 2021, Quinn 1995, Rateitschak 1971, Rosen 1981, RubelingJr 1979, Rudrud 1981, Sayers 1986, Sebregts 2020, See 2003, Shepherd 1971, Solberg 1972, Sugarman 1970, Sumner 3rd 1949, Tan 2000, Thayer 2022, Uthai 2021, Vavrina 2020, Yağci 2020, Yi 2013, Yüce 2013, Zeldow 1976.	Wrong study design (e.g., review article or case report), *n* = 74
Akabori 1984, Alcolea 2019, Alkumru 1985, Alonso-Navarro 2011, Arduino 1984, Buchner 1968, ChavarryAngulo 1973, Chikhani 2003, Gomesde 2012, Demner 1986, Doǧu 2009, EchevarríaMuro 1965a, Echeverriamuro 1965b, Fischer 2019, Fraccari 1988, Fröhlich 1966, Gastone 1983, Jimenez-CastellanosBallesteros 1991, Klewansky 1974, Kobayashi 1974, Korn 2005, Kotran 1981, Liem 2022, Mutschelknauss 1967, Orlova 2019, PascualHernandez 1988a, PascualHernandez 1988b, Permann 1987, Popov 1971, Popov 1987, Pourre 1985, Pourre 1986, Rateitschak 1966, Ring 1973, Romoli 2003, Spirgi 1970, Spirgi 1971, Su 2010, Tamdemir 2021, Teich 1979, Thom 1990, vanderMeulen 2000, vanderZaag 2000, Visscher 2000, Wang 1998, Yin 2004, Zhulev 1976, Zielinsky 1988.	Article not in English, *n* = 48
Ak 2009, Allen 2006, Bassett 2017a, Bassett 2017b, Chong 2007, Consumer reports on health 2022, Dull 2013, Goldstein 2012, Gunepin 2011, Hamilton 1986, Harnick 2000, Kishi 2007, Kucuk 2013, Malcmacher 2015, Malcmacher 2017, Nassif 1999, Orlova 2018, Patel 2022, Piekartz 2009, Piekartz 2022, Prater 2010, Quinn 2000, Rechmann 2018, Santos Miotto De Amorim 2015, Scharer 1974, Solberg 1973, Stein 1998, Thompson 1994, Valentin 1976, vanWaas 2001, VanZandijcke 1990, Velon 2015, Young 2009, Yurttutan 2018, Yurttutan 2019.	Not peer-reviewed full paper, *n* = 35
Ayer 1973, Ayer 1976, Baldini 2015, Cahlin 2019, Casas 1982, Cornellier 1982, Cruz-Reyes 2010, Dalewski 2015, Dalewski 2021, Diracoglu 2021, Gao 2019, Gholampour 2019, Grozev 1999, Guillot 2021, Heller 1975, Jardini 2006, Maeda-Iino 2020, Mkhitaryan 2020, Narita 2009, Ommerborn 2011, Peterson 1993, Rajpurohit 2010, Redaelli 2011, Saito‐Murakami 2020, Sullivan 2001, Treacy 1999, Trindade 2015, Yazici 2020, Yurttutan 2019, Yustin 1993	Not awake bruxism (AB) population, *n* = 30
Amsterdam 1992, Dias 2021, Emodi-Perlman 2021, Kef 2021, Pereira 2021, Wieselmann-Penkner 2001	Not AB management, *n* = 6
Begum 2019, Christensen 2000, James 2009, Lobbezoo 2018, Shatkin 1992	Review, *n* = 5
Alves 2009, CamposOrtega 1988, Garay 1975	Full text not found, *n* = 3
deLima 2021, Haggiag 2020, Lopes de Castro Bastos 2018	No relevant outcome, *n* = 3

### Description of studies

The nine included primary studies evaluated, in order of frequency of the investigated AB management strategy, biofeedback, guided music listening, habit reversal, medication, reminder prompts, and counselling with self-management (Table 3).

**Table 3:** Summary of extracted data for the included studies (*n* = 9)

First author Year Study setting Country	Study design Aim	Study sample Mean age (SD) range	Intervention(s)	Outcomes	Authors’ conclusion	Comments
Cioffi 2018 University clinic Canada	Case-control study To evaluate the effects of guided music listening (GML) on masticatory muscles and on the amplitude of wake-time tooth clenching in individuals with higher vs. lower frequencies of clenching episodes.	Pain-free university students HP = High parafunctional OBC-6 ≥9 10 (8 women) 21.4 yrs. (3.0) LP = Low parafunctional OBC-6 ≤3 11 (9 women) 22.6 yrs. (2.9)	Four sessions: 1. Relaxing music 2. Stressful music 3. Favourite music 4. Control (reading)	Rest EMG masseter: Highest stress task (HP+LP) (*P* <.001) Lowest during favourite music (LP), relaxing music (HP) (both *P* <.001). EMG clenching: Lower during favourite/stressful music compared with relaxing music (HP) (*P* < 0.05); lower during stressful music than reading (LP) (*P* =.001) Clenching episodes: NS frequency or duration	GML modulates masticatory muscle activity. The response to GML depends on the frequency of clenching and the type of music.	Intervention similar to that of Imbriglio 2020
Donnarumma 2022 University clinic Italy	Descriptive cohort study To evaluate the short-term effects of a standardized first-line non-invasive approach in women with chronic TMD myalgia and to test whether patients’ trait anxiety predicted response to treatment.	mTMD patients 14: 33.8 yrs. (11.1) 22–55 yrs.	1. Patient education 2. Self-monitoring 3. Avoiding oral behaviours 4. Jaw exercises and massage 5. Prevention	Masseter EMG activity reduced 30% (all *P* <.001). After 1 month: EMG total MVC decreased: mean (SEM) 1.51% (0.29) vs. 0.98% (0.29); contrast estimate: 0.50% (0.05) 95% CI: 0.44−0.62 (*P* <.001). EMG bruxism MVC decreased: mean (SEM): 28.26% (2.43) vs. 20.32% (2.60) MVC; contrast estimate: 8.04% (1.42) MVC; 95% CI: 5.25 to 10.84 (*P* <.001). Clenching episodes median (IQR): frequency decreased: 23 (31.2) vs. 9 (17.5) (*P* =.024); duration NS.	In the short term, FL-A reduces facial pain, masticatory muscle tenderness, and AB in women with chronic mTMD with low disability. A conservative management strategy should be prioritized for the initial management of these patients.	
Imbriglio 2020 University clinic Canada	Case control study To determine whether GML – a music intervention based on models of mood mediation and attention modulation – modulates masticatory muscle activity and AB in subjects with chronic painful muscular TMDs.	mTMD patients 14 women: median 39.5 yrs. (IQR 24.3) Controls 15 women: median 30.0 yrs. (IQR 3.5)	Four GML sessions: 1. Relaxing music 2. Stressful music 3. Favourite music, 4. Control (pink noise)	Rest EMG masseter (%MVC) mean (95% CI) affected in both groups compared with pink noise (all *P* <.001): mTMD: 2.2 (1.6−2.8); controls: 1.1 (0.5−1.7); - increased by stressful music: contrast estimate (95% CI); mTMD: +0.8 (0.7−0.8); controls: +0.3 (0.3−0.4); - decreased by relaxing music: mTMD: −0.4 (−0.5 to −0.4); controls: −0.3 (−0.4 to −0.3); - decreased by favourite music: mTMD: −0.5 (−0.6 to −0.5); controls: −0.5 (−0.5 to −0.4) EMG bruxism affected by group interaction (all *P* <.001). mTMD, compared with pink noise: 23.8 (16.0−31.6); contrast estimate (95% CI): increased by stressful music +10.2 (8.6−11.8), decreased by relaxing music −6.2 (−8.1 to −4.3) and favourite music −10.2 (−12.2 to −9.1) No effects in control group (*P* >.05). No effects for duration/frequency AB in either group.	In subjects with chronic mTMD, relaxing music and the individual’s favourite music decreased the muscular effort during spontaneous AB episodes by 26% and 44% (relative changes), respectively. In contrast, stressful music increased it by about 43%. Because of its positive effects on AB, GML with selected music could be a promising and non-invasive component of a multimodal approach for the management of chronic mTMD.	
Manns 1981 University clinic Chile	Descriptive cohort study Demonstrate the effectiveness of combined audiostimulation and EMG biofeedback in the treatment of bruxism and myofascial pain dysfunction syndrome.	TMD patients with bruxism 33 (26 women) 35 yrs., range 16−55 Group I: <1 year symptoms, *n* = 14 Group II: >1 year symptoms, *n* = 19	Audiostimulation combined with EMG biofeedback	Significant reduction in signs and symptoms* No difference between groups Rest EMG masseter decreased *bruxism, emotional tension, muscle fatigue, insomnia, headache, TMJ pain, masticatory muscles pain, neck muscle pain, articular clicking and mandibular deviation	We can conclude that the combination of audiostimulation and EMG biofeedback is an effective therapeutic method for reducing symptoms in each patient group. The groups did not show any significant differences when compared at the end of treatment. This means that the efficacy of this therapy is independent of the evolution of the syndrome.	Unclear reporting of the EMG data
Pfeiffer 2021 University clinic Germany	Case series To provide first clinical evidence that in-ear devices have a positive impact on relieving bruxism in patients.	TMD patients with bruxism 7 women Median 47.3 yrs., 23−64 yrs.	Personalized Cerezen in-ear device worn for increasing hours/day. Biofeedback through pressure on ear canal during closed jaw position.	Frequency of grinding reduced in some patients, but 5 of 7 discontinued treatment.	Despite the limited number of participants, the study reflects the potential of intra-aural devices as effective biofeedback devices in treating bruxism.	Large drop-out and no control group
Rosenbaum 1981 University clinic United States	Case series To extend the generality of habit-reversal to the problem of bruxism.	Individuals with bruxism 4 clients (2 women) 23−42 yrs.	Habit reversal by individual management	Bruxism reduced	The results of this study indicate that the habit-reversal technique (Azrin and Nunn, 1973, 1977) can be effective in decreasing and/or eliminating bruxism.	
Takeuchi-Sato 2020 University clinic Japan	Randomized controlled trial To evaluate the effectiveness of an email‐based recording and reminding system for limiting daytime non-functional tooth contact (nFTC) in patients with TMDs.	TMD patients 30 (17 women) 30.7 yrs. (8.7) e-CBT: 10 (7 women) 31.3 yrs. (10.6) s-CBT: 10 (5 women) 28.9 yrs. (8.2) controls: 10 (5 women) 32.1 yrs. (2.4)	1. e-mail-based CBT (eCBT) 2. CBT with sticky note reminders (sCBT) 3. Controls: instructed to keep teeth apart when noticing non‐functional contact.	Freq nFTC: Baseline (mean 95% CI) NS between groups (*p* = 0.33) 1. eCBT: 45.3 (36.1, 54.4); 2. sCBT: 44.1 (35.0, 53.1); 3. Ctrl: 44.4 (36.5, 52.3). After treatment: decreased e‐CBT and s‐CBT (*P* <.001); lower in e‐CBT than s‐CBT/control groups (*P* <.001); 1. eCBT: 16.5 (9.76, 23.3); 2. sCBT: 28.3 (22.0, 34.5); 3. Ctrl: 35.7 [29.4, 42.0]	The present findings suggest that our email‐based recording and reminding system may have the potential to effectively control daytime nFTC and could be an effective strategy for the management of TMDs	
Watanabe 2011 University clinic Japan	Randomized controlled trial Electromyogram (EMG) biofeedback training was performed to ascertain its effect on the regulation of daytime clenching behaviour.	Volunteers with pain/stiffness + daytime clenching 22 (11 women) 30.9 yrs. (5.6): 10 biofeedback (BF) 10 control (2 excluded)	BF: hearing-aid-shaped EMG recording/biofeedback apparatus (temporal muscle) 4 days, 5 hrs./day Days 2−3, BF group received clenching alert sound	Number clenching events: mean (SD) Day 1: BF 4.6 (2.5), control 4.6 (0.9) Day 4: BF 2.4 (1.7), control 4.4 (1.7); decreased in BF group (*P* < 0.05) NS control group		
Zandifar 2018 Hospital clinic Iran	Case series To find out from five case studies if low-dose Quetiapine manages the bruxism induced by the administration of selective serotonin reuptake inhibitors (SSRIs).	Patients with bruxism taking SSRIs 5 (4 women) median 28 yrs. (18−45 yrs.)	25−50 mg Quetiapine daily	Qualitative report of improvement in all five patients	Based on the results of the present study, low-dose Quetiapine can improve bruxism and mandibular dystonia, which are side effects of SSRIs.	

*BF* Biofeedback, *eCBT* email-based cognitive behavioural therapy, *sCBT* sticky-note-based cognitive behaviour therapy, *EMG* Electromyography, *FL-A* First-line non-invasive approach, *GML* Guided music listening,* HP* High parafunctional, *IQR* Interquartile range,* LP* Low parafunctional, *mTMD* Temporomandibular disorder myalgia, *MVC* Maximum voluntary contraction,* nFTC* non-functional tooth contact, *NS* Numerical rating scale, *OBC* Oral Behaviours Checklist, *SD* Standard deviation,* SSRI* Selective serotonin reuptake inhibitor, *TMDs* Temporomandibular disorders

Biofeedback was the most frequently proposed AB management strategy, being examined in three studies [52–54]. An intra-aural device was used in one study, but only two participants completed the trial [53]. In a EMG biofeedback investigation, the two days of the study protocol were insufficient to provide information on the control of daytime teeth clenching or on the duration of the effects [54]. In the third paper, audio stimulation and EMG biofeedback were jointly applied over multiple sessions, and a reduction in most symptoms was achieved after approximately eight sessions and symptom elimination after the last (14th) session [52]. Both studies based on EMG-biofeedback strategies were characterized by small sample sizes and short assessment periods, limited to a few days.

Guided music listening (GML) was the second most studied AB management strategy, being examined in two investigations [55, 56]. One evaluated the effect on the amplitude of AB clenching in a group of dental students with higher versus lower frequencies of clenching events [55]. The authors concluded that GML modulates masticatory muscle activity and that the response to GML depends on the frequency of clenching and the type of music. The second study sought to determine whether GML modulates masticatory muscle activity and AB in subjects with chronic TMD myalgia [56]. The conclusions suggest that GML could be useful in a multifaceted approach to AB management, but not as a primary or sole form of management.

The other AB management papers addressed other topics. In an investigation of habit reversal therapy [57], a technique described by Azrin and Nunn [58], procedures were developed to (a) increase the patient’s awareness of the occurrence of the habit, (b) interrupt the chain of behaviours terminating in the habit movement, (c) teach the patient a competing response opposite to the habit movement but involving the same muscle groups, and (d) eliminate social reinforcement of the habit. AB was decreased in all four patients and those results were maintained at 6 and 12 months [57]. Despite the adopted longitudinal study design, these findings are limited by the small sample size. A case report study conducted on five patients showed that Quetiapine, used in pharmacotherapy for major depressive disorder, tended to reduce AB events [59]. Another study utilised an email-based recording and reminding system with daily notifications to assess the effect of CBT and compare efficacy with that of conventional CBT and with the use of adhesive stickers as reminders [60]. Finally, the most recent primary study [61] assessed the effect of a first-line non-invasive approach, consisting of patient education and physical therapy, in terms of reducing pain and AB behaviour frequency. After one month of counselling and self-management measures, i.e., education and at-home exercises (general relaxation, jaw muscle massage and exercises, and thermal therapy), women with chronic masticatory myalgia displayed decreases in masseter muscle activity and clenching episodes.

Given the heterogeneity of included studies, meta-analysis was deemed not appropriate. Overall strength of evidence is summarised in a summary of evidence table (Table 4).

**Table 4:** Summary of evidence table (GRADE) based on included studies (*n* = 9)

Intervention	Number of studies, study design, participants (*n*)	Summary of findings	Certainty of evidence	Reasons for lowered certainty
CBT: Reminders emails/sticky notes	1[55], RCT (20 TMD patients, 10 controls)	Reduced non-functional tooth contact compared to controls	Low ¡¡**⊕⊕**	Only 1 study, small sample
Biofeedback	1[57], RCT (20 patients, 10 controls)	Reduced temporal EMG compared to controls	Low ¡¡**⊕⊕**	Only 1 study, small sample
Guided music listening	2[50, 51], case-control (50)	Reduced masseter EMG compared to controls	Low ¡¡**⊕⊕**	
Education + self management	1[56], cohort (14)	Reduced masseter EMG	Very low ¡¡¡**⊕**	Only 1 study, no control group, small sample
Audiostimulation + biofeedback	1[48], cohort (33)	Reduced masseter EMG	Very low ¡¡¡**⊕**	Only 1 study, no control group
Quetiapine	1[54], case series (5)	Qualitative report of improvement	Very low ¡¡¡**⊕**	Only 1 study, no control group, small sample
CBT: habit reversal	1[52], case series (4)	Reduced bruxism	Very low ¡¡¡**⊕**	Only 1 study, no control group, small sample
Intra-aural device	1[49], case series (7)	5 of 7 patients discontinued treatment	Very low ¡¡¡**⊕**	Only 1 study, no control group, small sample, large drop-out

*RCT* Randomised Controlled Trial, *TMD* Temporomandibular Disorders, *CBT* Cognitive Behavioural Therapy,* EMG* Electromyography

### Quality/risk-of-bias assessment

Three instruments were used for quality/risk-of-bias assessment depending on the study type. The Newcastle-Ottawa scale was used for non-randomized (i.e., case-control or cohort) studies, with cohort studies examined for outcome (Table 5) and case-control studies (Table 6) examined for exposure. The Joanna Briggs Institute Critical Appraisal Checklist was used for case-series (Table 7), and the RoB 2 scale for randomized controlled trials (Table 8). Most included studies demonstrated a moderate-to-high risk of bias, thereby limiting the strength of clinical recommendations The Cioffi et al., Imbriglio et al., Manns et al. and Pfeiffer et al., studies [52, 53, 55, 56] achieved moderate ratings, while four studies, Rosenbaum and Ayllon, Zandifar et al., Takeuchi-Sato et al., and Watanabe et al.,[54, 57, 59, 60] were rated as either poor or having a high risk of bias. The risk of bias for the Donnarumma et al. study [61], which examined counselling and self-management, was lower, but still limited by a relatively short data collection period of two months, only 20 min of EMG registration, and assessment only of clenching and not of other AB activities.

**Table 5:** Risk-of-bias assessment of included cohort studies using the Newcastle-Ottawa scale for cohort studies (*n* = 2)

First author, year	Selection						Comparability					Outcome							Total		
	S1	S2	S3	S4				C1	C2				O1	O2		O3					
Donnarumma 2022	«	NA	«	«				NA	NA				«	«		«				**6**	
Manns 1981	«	NA	«	«				NA	NA					«		«				**5**	

S1: Representativeness cohort; S2: Selection non-exposed cohort; S3: Ascertainment of exposure; S4: Outcome not present at start. C1: Age; C2: Other factors. O1: Assessment; O2: Length of follow-up; O3: Follow-up rate

Note, as per the definitions of the criteria for the Newcastle-Ottawa scale, scores for items S2, C1, and C2 are not applicable (NA) since the included studies did not involve non-exposed control groups

**Table 6:** Risk-of-bias assessment of included studies using the Newcastle-Ottawa scale for case-control studies (*n* = 5)

First author, year		Selection											Comparability							Exposure							Total				
	S1		S2		S3			S4				C1		C2				E1			E2		E3								
Cioffi 2018		«				«			«				«									«							**5**		
Imbriglio 2020		«				«							«									«		«					**5**		
Pfeiffer 2021*		«																											**1**		
Rosenbaum 1981*																													**0**		
Zandifar 2018*																													**0**		

S1: Definition cases; S2: Representativeness cases; S3: Selection controls; S4: Definition controls. C1: Age; C2: Other factors. E1: Assessment; E2: Same method for cases and controls; E3: Nonresponse rate

*Please note that per the definitions of the criteria in the Newcastle-Ottawa scale, studies without a control group cannot achieve scores for items S3, S4, C1, C2, E2, and E3

**Table 7:** Risk of bias assessment of included studies with the the Joanna Briggs Institute critical appraisal checklist for case-series (*n* = 3)

First author Year	1	2	3	4	5	6	7	8	9	10	Total
Pfeiffer 2021	N	Y	N	U	U	Y	Y	Y	Y	NA	50%
Rosenbaum 1981	N	U	N	U	U	Y	Y	Y	N	NA	30%
Zandifar 2018	N	N	U	U	U	Y	N	N	N	NA	10%

The 10 items were categorised as Yes (Y); No (N); Unclear (U); or Not Applicable (NA). Based on total Yes score (%) studies were classified as high (> 70%), moderate (50–70%), or low (< 50%) quality

**Table 8:** Risk-of-bias assessment of included randomized controlled trials (RCT) studies using RoB 2 (*n* = 2)

First author, year	Selection bias	Performance bias	Attrition bias		Detection bias		Reporting bias		Total
Takeuchi-Sato 2020	Low	Low	High		Unsure			Unsure	High
Watanabe 2011	Unsure	Unsure		Unsure		Unsure		Unsure	High

## Discussion

### Main findings

The main finding of the present review was that most strategies evaluated specifically for AB fall within the broad category of CBT. In addition, many articles identified in the literature search were excluded due to the lack of a clear distinction between sleep bruxism and AB or because they did not focus at all on an AB. Furthermore, included studies on AB were characterized by important methodological limitations related to small sample sizes, arbitrary strategies for instrumental assessment, lack of control groups, and cross-sectional design. Such important limitations affect the possibility of drawing clear conclusions on the long-term efficacy and causal relationships of the proposed treatment approaches.

Within these existing limitations, a crucial aspect highlighted in several studies is that patients with frequent AB behaviour are often not aware of its consequences^[57],60 61^. They typically seek help due to TMD pain and other orofacial pain conditions but are unaware of potential underlying causes. In this regard, some researchers have emphasised the importance of educating patients about the clinical repercussions of AB. The studies conducted by Donnarumma et al.[61] and by Rosenbaum and Ayllon [57], demonstrated the effectiveness of dedicating the initial part of CBT strategy to explaining to patients that the pain is likely due to AB-related overload of the temporomandibular joint structures. This aligns with recent findings on the relationship between pain and overload [62], and highlights the potential benefit of helping patients focus on the clinical implications of AB during the initial stages of CBT. Such recommendations must however be interpreted with caution, given that none of these studies evaluated potential placebo effects or the mechanism through which CBT contributed to the decrease of AB. Hence, it is not known if effects were obtained by the increased awareness, behavioural interruption, cognitive modulation of attention, reduction of anxiety or stress, increased contact with healthcare professionals, or a combination of these factors. Nevertheless, it is possible to hypothesize that CBT may contribute to reducing AB behavioural frequency through attention and self-regulation, without necessarily altering underlying neurobiological mechanisms.

In addition to this first step, several other authors have proposed different tools to help patients become aware of their AB behaviours during the day, ranging from the use of an index card where patients were asked to report their activities [57], to more sophisticated instruments such as an email system [60] or an EMG device emitting sounds whenever detecting AB activities [52]. Such strategies have been more rigorously defined and conceptualized by Bracci et al.[14] under the umbrella concept of the ecological momentary assessment (EMA) of AB. Indeed, one of the primary objectives of EMA, which is now part of the Standardized Tool for the Assessment of Bruxism (STAB) [12], is to enhance patients’ awareness of the frequency of their various oral behaviours.

Furthermore, Takeuchi-Sato et al.[60] demonstrated that combining EMA with education and advice during the initial counselling could further help patients. Examples are recommendations to avoid behaviours that may aggravate AB, such as excessive caffeine intake and irregular sleep schedules. Furthermore, daily email reminders were also used as an effective tool to motivate patients to keep their masticatory muscles in a relaxed state. In particular, they showed that the use of such daily reminders contributed to a significantly greater decrease in AB behaviours than did a single session of counselling [60]. A similar intervention, but with different tools, was proposed by Watanabe et al.[54], who, instead of adopting an email system, used surface EMG devices that emitted a sound whenever detecting a masticatory muscle activity related to AB. This approach also resulted in a significant decrease in AB compared with the control group that did not use the device. However, in both studies, the EMG assessment was limited to 5 h, which represents an important source of bias when evaluating the potential effect of CBT on the frequency of AB.

Despite these limitations, both interventions [52, 60] can be categorized as ecological momentary interventions (EMIs). Such strategies have been extensively documented in the psychology setting to treat conditions such as anxiety, depression, and substance abuse [63]. EMIs have also been advocated in the field of AB[64]; however, despite being a very promising strategy, their efficacy in terms of AB reduction and patient compliance has not yet been demonstrated in a large sample. Further investigations should explore the most effective method for delivering EMIs.

Two of the included studies also highlighted the effectiveness of incorporating exercises involving the masticatory muscles into daily CBT as a strategy to decrease muscle tension [57, 61]. Such exercises could help patients interrupt the chain of AB behaviours leading to overusing the orofacial musculoskeletal structures. In patients with TMD pain, such exercises could also help strengthen the muscles and promote their relaxation, helping relieve pain in accordance with the first-line conservative approach proposed in the IADR/INfORM guidelines as a good standard of TMD care [65]. Future research should aim to refine self-administered physical therapy techniques for patients who exhibit a higher frequency of AB. In this regard, it should be noted that the effect of these exercises was studied in a specific subgroup of patients with TMD pain [57, 61], limiting the external validity of the findings for other patient populations.

In the context of the association between AB and the psyche [20–24], the use of guided music listening (GML) as a method to decrease AB frequency has been proposed [55, 56]. The finding that music can influence clenching behaviour emphasizes the important role of the CNS in modulating bruxism. However, the effect was short-lived, and long-term maintenance as well as the method’s applicability in everyday clinical practice should still be evaluated. In these studies as well, the EMG assessments of AB were 20 min-periods or less, limiting the external validity of the findings [55, 56]. There are now portable EMG devices that can record a full day of muscle activity during wakefulness [66] and thereby provide a more comprehensive evaluation of the total amount and whole spectrum of AB-related jaw muscle activity. Indeed, with time, more refined and less invasive strategies for the EMG assessment of AB have emerged [19]. However, in contrast to the field of sleep bruxism, there is a lack of consensus with regard to standardised EMG assessment of AB[67]. To address this gap, the bruxism-work index (BWI) and the bruxism-time index (BTI) were proposed in the STAB [13]. Compared to the previous EMG criteria, these new indexes aim to measure the amount of bruxism-related masticatory muscle activity. The BWI is calculated by assessing the percentage of muscle work during bruxism-related masticatory muscle activity (MMA) compared to the potential work that could be exerted if the highest peak of power registered during the 24 h had been kept unvaried during all the bruxism episodes. Moreover, the BTI represents the percentage of time with bruxism-related MMA with respect to the total recording time [62, 66]. Among the unconventional strategies discussed in the literature, the use of an intra-aural device has been proposed [53], the function of which is to improve patients’ awareness upon activation. However, the device is activated only when the teeth come into maximum intercuspation, which is an important limitation, given that AB commonly involves masticatory muscle activity without tooth contact, namely mandible bracing [30]. Furthermore, the device users had high drop-out rates and side effects such as redness and sensation in the ear canal, reducing the method’s applicability. The use of the antipsychotic medication Quetiapine in patients with bruxism [59] was also studied in a case-series study, but no standardized assessment of AB was performed and no clear evidence emerged. As for now, the limited literature on the ability of drugs to decrease the AB frequency is limited to case reports [68], and given the existence of more conservative strategies without the risk for adverse effects, a pharmacologic approach cannot be advocated. Thus, based on these premises, the use of Quetiapine should not be considered a recommended strategy for the management of AB. Despite the detrimental role of psychological factors in the aetiology of AB[27, 69], it must be remarked that no studies have proposed administering tailored CBT in combination with support from a psychologist. Screening for anxiety and depression, as well as reduced coping skills, with the aim of phenotyping patients who require advanced psychological support, could also help dental practitioners better manage AB. Given that the many different strategies available for managing AB share many aspects, for practical purposes, the main basic principles can be summarized into a proposed “Five As” approach”:

- *Appraisal*: a clear explanation of the oral behaviour and the possible consequences of AB to the patient should be part of the initial process;
- *Awareness*: different techniques to increase awareness can be adopted, such as self-monitoring with the use of analogic or electronic devices that provide multiple feedback streams during the day;
- *Avoidance*: the patient should be informed of the possible modifiable risk factors and habits that can affect the frequency of AB, such as coffee intake, smoking, sleep deprivation, screen time, and drugs;
- *Advisement*: the patient should receive daily reminders of the importance of the first three steps; and.
- *Application*: the patient, upon receiving a reminder, should perform some physical exercises to release the muscle tension.

It is important to point out that this approach represents a conceptual proposal derived from the limited existing evidence available on the topic, rather than a data driven, validated clinical recommendation. The applicability of the Five As approach should therefore be evaluated and tested in individuals with different conditions to assess its efficacy in reducing the frequency of AB.

The main limitation of the available studies concerns the lack of EMG assessment of AB throughout the period of wakefulness. Such shortcomings have always characterized the research field of bruxism, due to the high costs and technical difficulties of adopting such monitoring protocols [66]. In the included studies, the monitoring periods were often limited to a few hours [52]or 20 minutes [56, 61]. Nevertheless, the research field now has the available tools to conduct longer investigations, and these tools have already found application in the field of orthodontics [17]. Given that currently no specific EMG criteria for the determination of different types of AB behaviours exist, a comprehensive assessment of AB should include the use of the specific self-report items taken from the STAB [13, 70], coupled with the adoption of the EMA strategies, allowing phenotyping different types of AB activities [14].

The second limitation is the small sample sizes that characterize most reviewed studies, which also lack a specific TMD diagnosis, further limiting the external validity in a specific subgroup of patients. The included nine primary studies were based on a total of only 165 patients, contributing to low evidence strength and limited generalisability of the findings [71]. The third aspect to consider is that that as AB tends to co-occur with sleep bruxism and that the two behaviours may influence each other [72, 73]; a comprehensive approach targeting both circadian manifestations could be more beneficial. Lastly, one of the shortcomings of the available studies is the lack of phenotyping of the AB spectrum of activities. The instrumental studies focused on teeth-clenching, which is just one type of AB behaviour, and no study evaluated the management of mandible bracing, a behaviour particularly prevalent in patients with TMD [30]. In this regard, future studies should focus on adopting a more specific approach when studying bruxism, rather than just using the generic term AB.

Further complicating the clinical scenario is the fact that bruxism, by being a behaviour, can tend to fluctuate naturally as well as due to non-specific treatment effects, such as placebo, that cannot be excluded and isolated in studies investigating the management of AB. In addition, the patients’ perception of being monitored can also have an impact on the reduction of AB behaviour, due to the Hawthorne effect [74], which has not been documented yet in the field of bruxism. None of the included studies could exclude such effects due to lack of proper, well-defined control groups, or due to the adoption of poorly characterized comparison groups. As a result, it remains difficult to disentangle intervention effects related to CBT from regression related to the mean, natural behavioural fluctuations, placebo effects, or patient expectancy effects. Future investigations should therefore adopt better-matched control groups.

Most studies included in this review were motivated by the resultant orofacial pain and its management or prevention, which could positively influence patients’ adherence to the therapy. Nevertheless, bruxism is a phenomenon that also has an impact on prosthetic restorations [75–77], so treatment strategies should be evaluated in prosthodontic patients as well.

Overall, the risk-of-bias assessment revealed medium or high risk of bias for most studies included in this review, often related to small patient numbers, short follow-up periods, and potentially confounding variables such as sleep bruxism. This review reinforces the paucity of research in this area. Given this, there is a great need for high-quality studies with a number of participants sufficient to conduct a controlled evaluation of the different AB management strategies. Moreover, there is a need for future studies to use consistent definitions and assessment methods for AB to enable reliable and valid comparisons across studies and settings. From our preliminary literature search, it emerged that not many publications examined AB management, so the study inclusion criteria were made as broad, and the exclusion criteria as narrow, as possible.

One strength of this review, is the comprehensive search in a large number of databases and other sources and the broad inclusion criteria set at the start. Except for one study [60], which compared two forms of AB management, no other studies compared their featured type of management with any other. Most of the primary studies had a high risk of bias, several studies had short follow-up periods, and the included studies used different criteria for defining AB.

The clinical implications of this review include the need to provide more comprehensive evidence-based clinical guidelines for both AB assessment and management. This will likely entail using a biopsychosocial model, as is the case for pain, to better understand AB prediction, onset, progression, and associated management.

## Conclusion

The results of this systematic review highlight the different CBT approaches available for managing AB. The existing literature on the management of AB is characterized by a lack of longitudinal studies, lack of control groups, small sample sizes and limitations related to the assessment of bruxism. Despite this, the available evidence suggests benefits of a behavioural approach to managing the frequency and consequences of awake bruxism.

## Supplementary Information

Supplementary Material 1

## Data Availability

Presented data from the primary studies are available by means of electronic bibliographic search. The full search strategy for all databases is presented in the Supplemental file.

## Abbreviations

AB: Awake bruxism
BWI: Bruxism-Work Index
CBT: Cognitive Behavioural Therapy
CNS: Central nervous system
EMA: Ecological Momentary Assessment
EMG: Electromyographic
EMI: Ecological Momentary Interventions
GML: Guided music listening
MMA: Masticatory muscle activity
STAB: Standardized Tool for the Assessment of Bruxism
TMD: Temporomandibular disorders
