# Supplementary material for: Management of awake bruxism: a systematic review
Source: BMC Oral Health. 2026 Feb 24;26:559. doi: 10.1186/s12903-026-07856-z (PMC13037066; doi:10.1186/s12903-026-07856-z)
Supplement: Supplementary file 1 — Supplementary Material 1 [file 12903_2026_7856_MOESM1_ESM.pdf]

Full search strategy

A. Medline, Embase, and CINAHL

1. “awake bruxism”
2. “awake clenching”
3. “awake parafunction”
4. “awake oral behavio*”
5. “awake oral habit*”
6. “diurnal bruxism”
7. “diurnal clenching”
8. “diurnal parafunction”
9. “diurnal oral behvio*”
10. “diurnal oral habit*”
11. “daytime bruxism”
12. “daytime clenching”
13. “daytime parafunction”
14. “daytime oral behavio*”
15. “daytime oral habit*”
16. Bruxism (advanced search limited to prevention, control, therapy)
17. 1 to 16 combined with OR
18. treatment
19. therap*
20. management
21. cure
22. intervention
23. surgery
24. 18 to 23 combined with OR
25. 17 and 24 combined with AND

B. Scopus, LILACS, Trip Medical and OpenGrey

“awake bruxism” OR “awake clenching” OR “awake parafunction” OR “awake oral behavio*” OR “awake oral habit*” OR “diurnal bruxism” OR “diurnal clenching” OR “diurnal parafunction” OR “diurnal oral behvio*” OR “diurnal oral habit*” OR “daytime bruxism” OR “daytime clenching” OR “daytime parafunction” OR “daytime oral behvio*” OR “daytime oral habit*” OR bruxism AND treatment OR therap* OR management OR cure OR intervention OR surgery

C. Google

1. "awake bruxism” and “management"

2. Screened first 14 pages sorted on relevance.
